# Socio-Educational Factors Associated With the Caries Index in Indigenous Amazonian Nationalities

**DOI:** 10.7759/cureus.86757

**Published:** 2025-06-25

**Authors:** Dennys Tenelanda Lopez, Kelly C Chavez, Daniel A Pallo, William Villa, Andrea C Merino

**Affiliations:** 1 School of Dentistry, National University of Chimborazo, Riobamba, ECU; 2 School of Forestry, Higher Polytechnic School of Chimborazo, Riobamba, ECU; 3 Cuatro Esquinas Health Center, Ministry of Public Health of Ecuador, Guaranda, ECU; 4 Calpi Health Center Type B, Ministry of Public Health of Ecuador, Riobamba, ECU; 5 Pediatric Dentistry, Hemisferios University, Riobamba, ECU

**Keywords:** caries, decayed missing and filled teeth (dmft), decayed missing and filled teeth (dmft) index, waorani, zaparo

## Abstract

Introduction: Dental caries is a chronic, multifactorial disease that causes demineralization and destruction of the tooth's hard tissues. Factors that impact caries formation include socio-educational aspects such as the level of study, economic status, and place of residence. Though there is some information about Indigenous people in Ecuador, there is scant literature concerning the Indigenous Amazonian nationalities like Waorani or Zaparo.

Objective: To describe the socio-educational aspects related to the caries level index of Ecuador's Waorani and Zaparo nationalities.

Methodology: This research was descriptive and cross-sectional, with a quantitative approach involving observation and survey techniques. The population consists of 89 patients of Waorani and/or Zaparo nationality. Based on the selection criteria, they were selected using non-probability convenience sampling.

Results: There was no difference between the two types of Amazonian nationality regarding the level of the caries index considered for the age group. Taking into account the indicators over which the caries index is measured (very low, low, moderate, and high) when associating them with the sex of the participants in this research, it was determined that only in the case of the mixed caries index (dmft / DMFT) there was a statistically significant association with sex. Additionally, there was a statistically significant association between the level of the DMFT index and patients' education level. Furthermore, a moderate positive correlation was observed between age and caries index levels across the three types of dentition.

Conclusion: Caries were present regardless of the type of Amazonian nationality, and their prevalence increased with the age of the population.

## Introduction

The oral cavity is a reflection of a person's health and well-being. Treatment for dental caries should be addressed at various levels, starting in early childhood. Effective oral hygiene, good nutrition, and ongoing follow-up care with specialists are essential during this stage. Caries are associated with some pathologies. Around 17.1% of obese child patients have a high prevalence of caries due to their diet rich in carbohydrates and sugars [1,2].

Parents require a certain level of knowledge about oral hygiene and a balanced diet to manage factors that predispose them to the development of cavities, such as diet and plaque control. It is essential that this information be passed on from parents to children. Studies have indicated that so-called anticipatory guidance is a health promotion model provided by health centers. Parents receive appropriate information, based on their children's developmental stage, regarding changes in their dentition, raising awareness of the importance of proper care and the consequences of developing cavities. Dental caries is related to people's general health. Childhood is the most vulnerable stage of a person's life, and the presence of severely advanced caries requires priority attention. The caries index is crucial because it provides information about the prevalence and severity of caries in a population or individual, allowing risk factors to be identified and evaluating the effectiveness of interventions. There is the DMFT caries index for permanent dentition and the dmft for deciduous dentition. Both are calculated by adding the number of decayed, missing, and filled teeth [2,3]. This indicator is part of a set of indicators designed to assess the status of dental caries [4].

In the context of oral health among indigenous peoples, a study conducted in Colombia by Ochoa-Acosta et al. shows that beliefs about oral health in indigenous communities are deeply rooted in their worldviews and traditional practices, which vary among ethnic groups. The Embera and Huitoto (indigenous nationalities of Colombia) perceive caries as the result of attacks by evil spirits. On the other hand, communities such as the Zenú and Kooris understand the disease as a natural imbalance. Oral hygiene practices include using natural elements such as salt, charcoal, and medicinal plants [5].

In a study by Isidro-Olán et al. focused on recognizing aspects of caries in Indigenous peoples of the Americas, a high prevalence of this disease was identified, influenced by multiple factors such as geographic isolation, social marginalization, and lack of access to adequate dental services. In communities such as the Maká in Paraguay, an extremely high rate of dental caries was reported, with a 93% prevalence among young people aged 15 to 19. Similarly, in the Mapuche community in Chile, a similar rate was observed, with an average of 13.5 teeth lost per adult. The situation is complicated by the lack of culturally appropriate preventive strategies and the poor integration of communities in the planning of state oral health policies, resulting in insufficient care and the persistence of high levels of caries in these vulnerable populations [6].

In the Ecuadorian context, according to the National Undersecretary of Public Health Surveillance [7]. In the results of the epidemiological situation room for the year 2022, dental caries was the cause of consultation of 4,242 patients, representing 0.18% of the burden on the health system, classifying the disease as one of the leading causes of morbidity at the national level. On the other hand, in the case of indigenous populations, specifically the Kichwa Saraguro ethnic group, a high prevalence of this disease was observed in 2018, mainly associated with factors such as dental plaque and dental calculus related to poor oral hygiene [8]. The Amazon region is home to several Indigenous nationalities, including the Waorani and Zaparo. There is limited information available about them regarding their oral health status. However, there are some issues related to their low socioeconomic status and lack of employment opportunities. These Indigenous nationalities are located in Pastaza province, specifically in Mera town and Shell location, where even the local health centers lack clear and accurate information about their general or oral health status [7].

The present research aimed to describe the socio-educational aspects related to the caries level index of Ecuador's Waorani and Zaparo nationalities.

## Materials and methods

This research employed a descriptive cross-sectional design with a quantitative approach. The study was non-experimental. In this research, observation and survey techniques were used. The Dental Clinical History form approved by the Ministry of Public Health of Ecuador [9] and an ad-hoc questionnaire on socio-educational aspects were used to collect data and establish the level of caries and socio-educational issues among patients from the Waorani and Zaparo communities.

The first data collection instrument categorizes the level of caries in the study population, considering the number of decayed, missing, and filled teeth (DMFT / dmft) pieces according to the type of dentition: high (4.5-6.5), medium (2.7-4.4), low (1.2-2.6), or very low risk (0.0-1.1) [4].

The second instrument, the ad-hoc questionnaire, was used to gather information about socio-educational aspects. It was explicitly designed for this study, considering indicators provided by the National Institute of Statistics of Ecuador [10] and the World Health Organization [11]. The questionnaire was composed of 15 items distributed in three main sections: (1) demographic data, such as age, gender, and place of residence; (2) socioeconomic indicators, such as self-reported income level and employment status; and (3) educational and cultural background, including the highest level of education achieved, native language, and type of Amazonian nationality (Waorani or Zapara). The instrument included both closed questions and open questions that allowed for a deeper contextual understanding. Content validity was evaluated by a panel of experts in education and intercultural studies, and a pilot test was conducted with 10 participants who shared similar characteristics to ensure clarity and cultural relevance. Based on the results of the pilot test, minor adjustments were made to enhance the understanding of the questions before their final application.

The DMFT/dmft (Decayed, Missing, and Filled Teeth) index was selected for this study due to its widespread use in epidemiological research, ease of application in field conditions, and its comparability with national and international oral health data. Although more detailed indices such as the International Caries Detection and Assessment System (ICDAS) or Pulpal Involvement, Ulceration, Fistula, Abscess (PUFA) provide greater diagnostic precision, the DMFT index was considered more suitable for the study context, which involved remote indigenous populations and limited clinical infrastructure. Furthermore, its simplicity allowed for efficient data collection without compromising the reliability of the caries prevalence estimation.

Data were obtained from medical records opened by the project Comprehensive Oral Health Program for the Waorani Nationality (Ecuador), Resolution No. 40-IRB-1-3-2023. The population consisted of 89 Waorani and Zaparo nationality patients participating in the Comprehensive Oral Health Program for the Waorani Nationality (Ecuador) project of the Dentistry in Action research group. They were selected using a non-probability convenience sampling based on the selection criteria. This included individuals of Waorani and Zaparo nationalities who were part of the research project, had signed the informed consent form (or whose parents had provided consent, in the case of minors), had no mental health conditions, agreed to participate for the duration of the study, and were considered non-vulnerable. This research was conducted in 2024 within the town of Mera in Pastaza province.

The dental records were completed by previously calibrated professionals to ensure consistency and reliability in data collection. Calibration was carried out through theoretical and practical training sessions using standardized DMFT index criteria. Inter-examiner agreement was assessed, yielding a kappa coefficient above 0.80.

The results were presented through descriptive statistics. According to the type of variables, Spearman’s rho and chi-square tests were used to check whether there was an association between the variable socio-educational aspects and the DMFT, dmft, or DMFT/dmft caries index [12]. The collected data were analyzed using the SPSS statistical program, version 28 (IBM Corp., Armonk, NY).

## Results

Regarding the age distribution among two Amazonian Indigenous nationalities, the majority of the Zaparo and Waorani groups comprised children, with 17 (56.7%) and 29 (49.2%), respectively. For the Zaparo, the second largest group consisted of young adults (n=7; 23.3%), with the remaining age groups containing two participants (6.7%) in each case. Among the Waorani, the age distribution was as follows: 13 individuals in early adulthood (22.0%), 12 in middle adulthood (20.3%), five adolescents (8.5%), and no elderly individuals (Table 1).

**Table 1:** Age distribution among the Amazonian Indigenous nationalities f: frequency

Indigenous Nationality		Childhood	Adolescence	Adulthood			Total
				Early Adulthood	Middle Adulthood	Late Adulthood	
Zaparo	f	17	2	7	2	2	30
	% within Indigenous nationality	56.70%	6.70%	23.30%	6.70%	6.70%	100.00%
	% within Age	37.00%	28.60%	35.00%	14.30%	100.00%	33.70%
	% of the total	19.10%	2.20%	7.90%	2.20%	2.20%	33.70%
Waorani	f	29	5	13	12	0	59
	% within Indigenous nationality	49.20%	8.50%	22.00%	20.30%	0.00%	100.00%
	% within Age	63.00%	71.40%	65.00%	85.70%	0.00%	66.30%
	% of the total	32.60%	5.60%	14.60%	13.50%	0.00%	66.30%
Total	f	46	7	20	14	2	89
	% within Indigenous nationality	51.70%	7.90%	22.50%	15.70%	2.20%	100.00%
	% within age	100.00%	100.00%	100.00%	100.00%	100.00%	100.00%
	% of the total	51.70%	7.90%	22.50%	15.70%	2.20%	100.00%

Regarding gender distribution, it is observed that in both the Zaparo and Waorani communities, women constituted the majority of the study population. Among the Zaparo, 18 (60.0%) are women and 12 (40.0%) are men. Among the Waorani, 39 (66.1%) were women, and 20 (33.9%) were men. Overall, 57 (64.0%) of the total population were female, while 32 (36.0%) were male, indicating a predominance of women in these Amazonian indigenous communities (Table 2).

**Table 2:** Sex distribution among the Amazonian Indigenous nationalities

Indigenous Nationality		Sex		
		Male	Female	Total
Zaparo	f	12	18	30
	% within Indigenous nationality	40.00%	60.00%	100.00%
	% within sex	37.50%	31.60%	33.70%
	% of the total	13.50%	20.20%	33.70%
Waorani	f	20	39	59
	% within Indigenous nationality	33.90%	66.10%	100.00%
	% within sex	62.50%	68.40%	66.30%
	% of the total	22.50%	43.80%	66.30%
Total	f	32	57	89
	% within Indigenous nationality	36.00%	64.00%	100.00%
	% within sex	100.00%	100.00%	100.00%
	% of the total	36.00%	64.00%	100.00%

Regarding economic status, most individuals in both Amazonian indigenous communities fell into the lower economic status category. In the Zaparo community, 29 (96.7%) reported a lower economic status, while only one (3.3%) had a middle economic status. In the Waorani community, 54 (91.5%) of individuals also had a lower economic status, with five (8.5%) having a middle economic status. Around 93.3% (n=83) of the population had a lower economic status, while only six (6.7%) belonged to the middle class (Table 3).

**Table 3:** Economic status levels among the Amazonian Indigenous nationalities f: frequency

Indigenous Nationality		Economic Status		Total
		Lower	Middle Class	
Zaparo	f	29	1	30
	% within Indigenous nationality	96.70%	3.30%	100.00%
	% within economic condition	34.90%	16.70%	33.70%
	% of the total	32.60%	1.10%	33.70%
Waorani	f	54	5	59
	% within Indigenous nationality	91.50%	8.50%	100.00%
	% within economic condition	65.10%	83.30%	66.30%
	% of the total	60.70%	5.60%	66.30%
Total	f	83	6	89
	% within Indigenous nationality	93.30%	6.70%	100.00%
	% within economic condition	100.00%	100.00%	100.00%
	% of the total	93.30%	6.70%	100.00%

Regarding education level, many individuals in both Amazonian Indigenous communities did not receive formal schooling. In the Zaparo community, 21 (70.0%) individuals have no registered level of formal education, followed by five (16.7%) who completed secondary education, three (10.0%) with primary education, and only one (3.3%) with a bachelor's degree. In the Waorani community, 29 (49.2%) also did not receive formal education, while 16 (27.1%) completed primary education, nine (15.3%) completed secondary education, and five (8.5%) completed a bachelor's degree. Overall, 50 (56.2%) of the total population did not receive formal education, 19 (21.3%) completed primary education, 14 (15.7%) completed secondary education, and six (6.7%) have a university degree (Table 4).

**Table 4:** Education levels among the Amazonian Indigenous nationalities f: frequency

Indigenous Nationality		Level of Education				Total
		Not Applicable	Elementary	Highschool	Graduated	
Zaparo	f	21	3	5	1	30
	% within Indigenous nationality	70.00%	10.00%	16.70%	3.30%	100.00%
	% within education level	42.00%	15.80%	35.70%	16.70%	33.70%
	% of the total	23.60%	3.40%	5.60%	1.10%	33.70%
Waorani	f	29	16	9	5	59
	% within Indigenous nationality	49.20%	27.10%	15.30%	8.50%	100.00%
	% within education level	58.00%	84.20%	64.30%	83.30%	66.30%
	% of the total	32.60%	18.00%	10.10%	5.60%	66.30%
Total	f	50	19	14	6	89
	% within Indigenous nationality	56.20%	21.30%	15.70%	6.70%	100.00%
	% within education level	100.00%	100.00%	100.00%	100.00%	100.00%
	% of the total	56.20%	21.30%	15.70%	6.70%	100.00%

The Zaparo and Waorani communities have different levels of dental caries concerning the dmft index. In the Zaparo community, four (50.0%) individuals have a moderate dmft level, while two (25.0%) have a low level, and one individual each (12.5%) falls into the very low and high categories. In the Waorani community, the distribution shows that five (35.7%) individuals have a high dmft level, followed by four (28.6%) with a moderate level, three (21.4%) with a very low level, and two (14.3%) with a low level. Overall, eight (36.4%) of the total population have a moderate dmft level, six (27.3%) have a high level, and four individuals (18.2%) each in both low and very low levels (Table 5).

**Table 5:** dmft index levels among the Amazonian Indigenous nationalities

Indigenous Nationality		dmft level				Total
		Very low	Low	Moderate	High	
Zaparo	f	1	2	4	1	8
	% within Indigenous nationality	12.50%	25.00%	50.00%	12.50%	100.00%
	% within dmft level	25.00%	50.00%	50.00%	16.70%	36.40%
	% of the total	4.50%	9.10%	18.20%	4.50%	36.40%
Waorani	f	3	2	4	5	14
	% within Indigenous nationality	21.40%	14.30%	28.60%	35.70%	100.00%
	% within dmft level	75.00%	50.00%	50.00%	83.30%	63.60%
	% of the total	13.60%	9.10%	18.20%	22.70%	63.60%
Total	f	4	4	8	6	22
	% within Indigenous nationality	18.20%	18.20%	36.40%	27.30%	100.00%
	% within dmft level	100.00%	100.00%	100.00%	100.00%	100.00%
	% of the total	18.20%	18.20%	36.40%	27.30%	100.00%

Regarding the DMFT index, both Amazonian indigenous communities have a high prevalence of high caries levels, but with notable differences. In the Zaparo community, seven (38.9%) individuals have both moderate and high DMFT levels, while four (22.2%) have a low level, and no cases have been recorded at a very low level. On the other hand, in the Waorani community, 17 (53.1%) have a high DMFT level, followed by seven (21.9%) at a low level, five (15.6%) at a moderate level, and three (9.4%) at a very low level. Overall, 24 (48.0%) of the total population has a high DMFT level, 12 (24.0%) at a moderate level, 11 (22.0%) at a low level, and three (6.0%) at a very low level (Table 6).

**Table 6:** DMFT index levels among the Amazonian Indigenous nationalities

Indigenous Nationality		DMFT level				Total
		Very low	Low	Moderate	High	
Zaparo	f	0	4	7	7	18
	% within Indigenous Nationality	0.00%	22.20%	38.90%	38.90%	100.00%
	% within DMFT level	0.00%	36.40%	58.30%	29.20%	36.00%
	% of the total	0.00%	8.00%	14.00%	14.00%	36.00%
Waorani	f	3	7	5	17	32
	% within Indigenous Nationality	9.40%	21.90%	15.60%	53.10%	100.00%
	% within DMFT level	100.00%	63.60%	41.70%	70.80%	64.00%
	% of the total	6.00%	14.00%	10.00%	34.00%	64.00%
Total	f	3	11	12	24	50
	% within Indigenous Nationality	6.00%	22.00%	24.00%	48.00%	100.00%
	% within DMFT level	100.00%	100.00%	100.00%	100.00%	100.00%
	% of the total	6.00%	22.00%	24.00%	48.00%	100.00%

In the Zaparo community, two (50.0%) individuals have a low level of caries in mixed dentition, followed by one (25.0%) with very low and moderate levels, respectively. No cases were recorded at the high level. In the Waorani community, most individuals have low levels of caries in mixed dentition, with five (38.5%) having a low level, three (23.1%) having a moderate level, four (30.8%) having a high level, and one (7.7%) having a very low level. Overall, 41.2% (n=7) of the total population has a low level of caries in mixed dentition, four (23.5%) have both moderate and high levels, and two (11.8%) have very low levels (Table 7).

**Table 7:** DMFT/dmft levels in mixed dentition among Amazonian Indigenous nationalities

Indigenous Nationality		Mixed Level (DMFT/dmft) index				Total
		Very Low	Low	Moderate	High	
Zaparo	f	1	2	1	0	4
	% within Indigenous nationality	25.00%	50.00%	25.00%	0.00%	100.00%
	% within the mixed level	50.00%	28.60%	25.00%	0.00%	23.50%
	% of the total	5.90%	11.80%	5.90%	0.00%	23.50%
Waorani	f	1	5	3	4	13
	% within Indigenous nationality	7.70%	38.50%	23.10%	30.80%	100.00%
	% within the mixed level	50.00%	71.40%	75.00%	100.00%	76.50%
	% of the total	5.90%	29.40%	17.60%	23.50%	76.50%
Total	f	2	7	4	4	17
	% within Indigenous nationality	11.80%	41.20%	23.50%	23.50%	100.00%
	% within the mixed level	100.00%	100.00%	100.00%	100.00%	100.00%
	% of the total	11.80%	41.20%	23.50%	23.50%	100.00%

There was no difference between the two types of Amazonian nationality regarding the level of the caries index considered for the age group. Taking into account the indicators over which the caries index is measured (very low, low, moderate, and high) when associating them with the sex of the participants in this research, it was determined that only in the case of the mixed caries index (dmft / DMFT) there was a statistically significant association with sex. As shown in the table, there was a statistically significant association between the level of the DMFT index and patients' education level (Table 8).

**Table 8:** Association between caries indices (dmft, DMFT, and mixed dentition index) and socio-educational factors Significance: * p < 0.05.

Caries Index	Zaparo Nationality			Waorani Nationality			Sex			Level of Education			Economic Status			N
																		p	Degrees of freedom	Effect size	p	Degrees of freedom	Effect size	p	Degrees of freedom	Effect size	p	Degrees of freedom	Effect size	p	Degrees of freedom	Effect size	
																	dmft	0.614	1	0.12	0.234	1	0.25	0.177	1	0.29	0.66	3	0.08	0.322	1	0.21	89
DMFT	0.375	1	0.18	0.455	1	0.15	0.636	1	0.09	0.017*	3	0.42	0.646	1	0.08	89
Mixed caries index (dmft/DMFT)	0.126	1	0.32	0.442	1	0.16	0.011*	1	0.48	0.098	3	0.35	0.401	1	0.17	89

When relating the caries index of the three types of dentitions with age, there was a statistically significant p-value (0.010) association with a moderate positive correlation. The older the age, the higher the caries index (Table 9).

**Table 9:** Association between caries indices (dmft, DMFT, and mixed dentition index) and age Significance: * p < 0.05.

Caries Index	Age		
	Correlation Coefficient	Sig (Bilateral)	N
Dmft index	0.331	0.218	89
DMFT Index	0.549	0.010*	89
Mixed dentition caries index (dmft/DMFT)	-0.366	0.148	89

## Discussion

Regarding the participants' gender in this research, the results show that the female gender is predominant in both the Zaparo and Waorani Amazonian Indigenous nationalities. These results are similar to those presented by Scazza and Nenquimo [13], who conducted a study in a Waorani group and determined the female predominance. Similarly, Hussain et al. [14] studied aspects of a Canadian Indigenous community where women outnumbered men. On the other hand, some data are opposite to those presented in this manuscript. For example, Valdivieso et al. [15] worked with an Indigenous Mayan population where the male gender prevailed, as did Aamodt et al. [16], who presented the same frequency. Finally, the same number of men and women were included in a study by Cuenca et al. [17].

Concerning the economic level, it was observed that both the populations of Zaparo and Waorani belong to a low economic stratum. Along the same lines, studies by Valdivieso et al. [15] and the Technical Secretariat of the Special Amazon Territorial District [18] have mentioned that this territory has the highest level of extreme poverty and numerous unmet needs. Similar data to that of neighboring countries, often referred to as developing countries, is available. For instance, research conducted by Calderón et al. [19] in a Colombian Indigenous community provides a relevant example. In contrast to what was mentioned above, it is observed that in first-world countries such as Australia, the economic stratum of their Indigenous populations is higher, as reported in research by Islam et al. [20], where the majority belong to the middle economic stratum.

The data indicated that the majority of the study population fell within the childhood age range. This percentage is similar to that reported by Cuenca et al. [17] and the results of the Ecuador census published by the National Institute of Statistics and Censuses [21] in Pastaza province, where it is specified that 44.6% of the population is Indigenous, with a higher population density among children under 14 years of age. Likewise, Valdivieso carried out a study in which the highest percentage of the age group belonged to those aged 0-20 years among the Amazonian Indigenous nationalities of Ecuador. The information mentioned above can be explained because the mortality rate during the 2020 pandemic was higher than 90% in the 21- to 65-year-old age group. On the other hand, in a study in Mexico, most participants were between 14 and 20 years of age [16].

Finally, it was observed that in both the Zaparo and the Waorani, the majority had primary education, a result corroborated by the Comprehensive Plan for the Amazon [18], which highlights that in the Amazon, there are low levels of schooling in the population, with an illiteracy rate of 1.36%. In this sense, the information presented by Salinas et al. [22] showed that 42.7% of adults had completed their primary education, and 27% had completed secondary education in their Indigenous population in the Amazon. These results are supported by the research carried out by Hohenthal et al. [23], who noted that in Pastaza, the closed units were primarily primary schools. Still, the increasing dropout rates at this level have probably affected the number of students entering secondary, upper secondary, and higher education. In contrast, it can be observed that in developed countries such as Canada, there is a massive gap concerning the level of education of its Indigenous communities, as reported by Hussain et al. [14], where the majority of the Indigenous population had a fourth level of education.

It was recognized that the Waorani population mostly presented a high dmft index (35.7%), a high DMFT index (53.1%), and a low caries index in mixed dentition (38.5%). On the other hand, the Zaparos mostly presented a moderate dmft index (50.0%), a high DMFT index (38.9%), and a low caries index in mixed dentition (50.0%). Similar results were reported by Rojas et al. [24], where variation in the DMFT and dmft indices was observed among the Amazonian Indigenous populations of Venezuela, ranging from moderate to high. A similar finding was reported in a research study conducted in Peru by Aquino-Canchari et al. [25], who found that the majority of inhabitants in Indigenous communities had a high DMFT index. Results are consistent with a systematic review of dental caries in Indigenous peoples of South America, which considered studies from 1964 to 2018 [26]. The realities of developing countries contrast with the caries index of Indigenous populations in developed countries, such as Australia, where these populations experience caries but at lower rates, considering that public health policies contribute significantly to the health of their Indigenous populations [27].

Considering the type of Amazonian Indigenous nationality, in this investigation, there were no statistically significant differences between the Waorani and the Zaparos in terms of the caries index they presented. Similarly, a retrospective study by Ortiz De Orué-Ninantay et al. [28] in two Indigenous Inca populations (Sacsayhuamán and Machupicchu) found differences in the frequency of dental caries and their location. Still, no differences were found in terms of severity. These results differ from those of the study by Rebelo et al. [29] on Brazilian Indigenous nationalities, which found significant differences in the prevalence of dental caries by place of residence.

In this study, the caries index (DMFT) showed a significant relationship with age. This result is consistent with the report by Alves [30] in an Indigenous Guaraní population (Brazil), who determined that the older the age, the greater the dental problems. There were other similar findings in which a statistically significant association was established between these two variables, such as those presented by Zambrano et al. [31], Curtis et al. [32], and Rojas et al. [24]. In the first case, a study was carried out among Indigenous residents of Corozal and Maniapure in Venezuela. In the second study, the research was conducted in three Indigenous communities located in the mountains. Lastly, the study focused on Indigenous residents of the Autana municipality in Venezuela.

On the other hand, in this investigation, the dmft and DMFT indices did not show a statistically significant association with sex. This result was similar to that of Rojas et al. [23], who determined no relationship between dental caries in Indigenous Autana municipality, Venezuelan residents, and sex. In this sense, both investigations are consistent with the study reported by Aamodt et al. [16], who conducted their research in an Indigenous population in Chiapas, Mexico, and obtained similar findings. However, a study with opposite results was found, published by Zambrano et al. [31], who researched Indigenous residents in Corozal and Maniapure, Venezuela, finding a relationship between the variables of caries index and sex.

Finally, UNESCO [33] states that education is essential in preventing and controlling diseases, as it forms the basis for patients' behavior when faced with a health problem of their own or that of their family. This study found a correlation between the participants' educational level and the caries index. This result is aligned with those presented by Chinnakotla et al. [34], Ellakany et al. [35], and Holve et al. [36], who found the same findings when associating the two study variables in question. No studies were found in Indigenous populations that reported results contradicting this research.

Some limitations were identified during this study. There was not much existing reliable research about the Amazon Indigenous nationalities of Waorani and Zaparo to compare these current results and analyze differences or similarities concerning the variables taken into account in this study, such as age, sex, economic status, level of education, and type of Amazon nationality, the DMFT, dmft, or DMFT/dmft caries index.

The research was conducted with a limited population due to the local authorities' lack of facilities. Longitudinal future studies should include a larger population and analyze additional variables, such as dental plaque, other oral pathologies, dietary habits, and oral hygiene practices. It is also important to complement future studies with other indices that allow for determining the severity and clinical impact on patients, such as the ICDAS or PUFA indexes.

## Conclusions

The results of this study reveal how social vulnerability and limited access to education and healthcare shape oral health outcomes in the Waorani and Zaparo Indigenous populations. Rather than merely reflecting isolated clinical data, the patterns observed--such as high caries prevalence and associations with age, gender, and educational level--point to deeper systemic challenges. These findings underscore the importance of viewing oral health within the broader context of social determinants and highlight the need for targeted public health strategies that are culturally appropriate and community-focused. By documenting the oral health status of two underrepresented Amazonian nationalities, this study contributes to addressing a significant gap in epidemiological data and supports efforts to reduce health inequities among Indigenous communities

Although this study was primarily designed to assess the caries index and socio-educational characteristics among the Waorani and Zaparo nationalities, its findings have broader public health implications. Indigenous populations in the Amazon region often face structural inequalities in access to oral health services, education, and preventive care. By highlighting patterns of dental caries and their association with age, gender, and educational level, this study contributes to the existing body of knowledge and underscores the urgent need for culturally appropriate, community-based oral health interventions in these underserved groups. Thus, the correlation between methodology and objective is not only internally valid but also relevant to addressing a wider gap in oral health equity.

## References

[1] Aldana Salguero JE, Silva Menjívar AE (2022). Relationship between dental caries and body mass index in children. Alerta.

[2] González A, González B, González E (2013). Dental health: relationship between dental caries and food consumption. Nutr Hosp.

[3] Mayor Hernández F, Pérez Quiñones JA, Cid Rodríguez MC, Martínez Brito I, Martínez Abreu J, Moure Ibarra MD (2014). Dental caries and its interrelation with some social factors [article in Spanish]. Rev Med Electrón.

[4] (2025). WHO: Mean number of Decayed, Missing, and Filled Permanent Teeth (mean DMFT). https://www-who-int.translate.goog/data/gho/indicator-metadata-registry/imr-details/3812?_x_tr_sl=en&_x_tr_tl=es&_x_tr_hl=es&_x_tr_pto=tc.

[5] Ochoa-Acosta EM, Patiño-Gutiérrez K, Pérez-Suescún CA, Lambraño-Escobar LF, Sierra-Caro E (2015). Cultural traditions and oral care practices among the Zenú indigenous people in Sucre, Colombia [article in Spanish]. Rev Nac Odontol.

[6] Isidro-Olán LB, Estrella-Castillo DF, Vega-Lizama EM, Rueda-Ventura MA, Rubio-Zapata HA (2022). Influence of social determinants on oral health in indigenous populations of the Americas. Literature review [article in Spanish]. Odontol Sanmarquina.

[7] (2024). Ministry of Public Health of Ecuador. Epidemiological Situation Room. https://www.salud.gob.ec/wp-content/uploads/2024/04/Sala-de-Situacion-DNVE_2022.pdf.

[8] Centeno Dávila MC, Lazo Aguirre KN, Pérez Mora AP, Jiménez Romero MN (2023). Factores modificables y no modificables asociados a la enfermedad periodontal. Estudio en la etnia Kichwa Saraguro, Ecuador. Sociedad del Conocimiento [book in Spanish].

[9] (2025). Ministry of Public Health of Ecuador. Manual for Use of Form 033 [source in Spanish]. https://web.archive.org/web/20220615145659/https://aplicaciones.msp.gob.ec/salud/archivosdigitales/documentosDirecciones/dnn/archivos/HISTORIA%20CL%C3%8DNICA%20%C3%9ANICA%20DE%20SALUD%20BUCAL.pdf.

[10] (2025). INEC Socioeconomic Level Stratification Survey. https://www.ecuadorencifras.gob.ec/encuesta-de-estratificacion-del-nivel-socioeconomico/.

[11] (2025). Euroinnova Etapas de la vida por edad [source in Spanish]. https://www.euroinnova.com/blog/etapas-de-la-vida-por-edad.

[12] Reguant-Álvarez M, Vilà-Baños R, Torrado-Fonseca M (2018). La relación entre dos variables según la escala de medición con SPSS [article in Spanish]. REIRE Revista d’Innovació i Recerca en Educació.

[13] Scazza M, Nenquimo O (2021). From Spears to Maps: the Case of Waorani Resistance of their Right to Prior Consultation. https://www.iied.org/sites/default/files/pdfs/2021-02/12612IIED.pdf.

[14] Hussain A, Jaimes SB, Crizzle AM (2021). Predictors of self-rated oral health in Canadian Indigenous adults. BMC Oral Health.

[15] Valdivieso G, Stefos E, Lalama R (2017). The Ecuadorian Amazon: A data analysis of social and educational characteristics of the population. Rev Eur Stud.

[16] Aamodt K, Reyna-Blanco O, Sosa R, Hsieh R, De la Garza Ramos M, Garcia Martinez M, Orellana MF (2015). Prevalence of caries and malocclusion in an indigenous population in Chiapas, Mexico. Int Dent J.

[17] Cuenca K, Vélez E, Solano P, Salto D, Bustamante J (2020). Dental caries in students from the rural parishes of Ingapirca and Ducúr, Ecuador. Rev Eur Stud.

[18] Secretaría Técnica de la Circunscripción Territorial Especial Amazónica (2021). Plan Integral para la Amazonía. Plan Integral para la Amazonía [book in Spanish].

[19] Calderón M, Contreras T, García M, Lozano M (2021). Social determinants and their relationship with dental caries in a school-age population aged 5 to 12. https://repository.usta.edu.co/server/api/core/bitstreams/97ca9c81-de33-46f4-bb03-3f73ab60de9a/content.

[20] Islam MI, Chadwick V, Esgin T, Martiniuk A (2022). Bullied because of their teeth: evidence from a longitudinal study on the impact of oral health on bullying victimization among Australian Indigenous children. Int J Environ Res Public Health.

[21] Instituto Nacional de Estadística y Censos (2022). Censo Ecuador Cuenta conmigo. https://www.censoecuador.gob.ec/wp-content/uploads/2024/05/Presentacion_Nacional_2da_entrega.pdf.

[22] Salinas Castro V, Bilsborrow RE, Gray C (2020). Socioeconomic changes in the 21st century in Amazonian indigenous populations: current challenges. Estud Demogr Urbanos Col Mex.

[23] Hohenthal J, Minoia P (2022). Territorial and mobility justice for Indigenous youth: accessing education in Ecuadorian Amazonia. Mobilities.

[24] Rojas F, Cedeño J, Rivera M, Acevedo A (2018). Prevalence of dental caries in indigenous populations of the Autana Municipality, Amazonas State, Venezuela [article in Spanish]. Odous Científica.

[25] Aquino-Canchari CR, Caro-Aylas HW, Crisol-Deza DA, Zurita-Borja JL, Barrientos-Cochachi JE, Villavicencio-Caparo E (2019). Clinical epidemiological profile of oral health in Peruvian native communities [article in Spanish]. Rev Habanera Ciencias Médicas.

[26] Nath S, Poirier BF, Ju X, Kapellas K, Haag DG, Ribeiro Santiago PH, Jamieson LM (2021). Dental health inequalities among indigenous populations: a systematic review and meta-analysis. Caries Res.

[27] Australina Goverement (2024). Australian Institute of Health and Welfare. Aboriginal and Torres Strait Islander Health Performance framework. Oral health. https://www.indigenoushpf.gov.au/measures/1-11-oral-health.

[28] Ortiz De Orué-Ninantay D, Villavicencio-Caparó E, Peña-Alegre MC (2024). Dental caries in two Inca populations. A cross-sectional study [article in Spanish]. Rev Cient Odontol (Lima).

[29] Rebelo Vieira JM, Pereira JV, Sponchiado Júnior EC (2023). Prevalence of dental caries, periodontal disease, malocclusion, and tooth wear in indigenous populations in Brazil: a systematic review and meta-analysis. Braz Oral Res.

[30] Alves Filho P, Santos RV, Vettore MV (2014). Factors associated with dental caries and periodontal diseases in Latin American indigenous peoples: a systematic review [article in Portuguese]. Rev Panam Salud Publica.

[31] Zambrano JG, Urbina-Blanco VH, Esis-Villarroel IM, Montero M, Acevedo A (2014). Dental caries pattern in indigenous peoples residing in Corozal, Maniapure, Bolivar State, Venezuela [article in Spanish]. Acta Odontológica Venezolana.

[32] Curtis D, Ortega F, Monar J, Bay R, Eckhart S, Thompson P (2017). Assessing self-reported oral health status of three Andean indigenous communities in Ecuador. J Int Oral Heal.

[33] (2025). UNESCO Salud y Educación [source in Spanish]. UNESCO. Health and Education [Internet.

[34] Chinnakotla B, Susarla SM, Mohan DC, Turton B, Husby HM, Morales CP, Sokal-Gutierrez K (2022). Associations between maternal education and child nutrition and oral health in an indigenous population in Ecuador. Int J Environ Res Public Health.

[35] Ellakany P, Madi M, Fouda SM, Ibrahim M, AlHumaid J (2021). The effect of parental education and socioeconomic status on dental caries among Saudi children. Int J Environ Res Public Health.

[36] Holve S, Braun P, Irvine JD, Nadeau K, Schroth RJ (2021). Early childhood caries in indigenous communities. Pediatrics.

